# Effects of Digital Health Literacy Program on Sufficient Health Behavior Among Thai Working-Age People With Risk Factors for Noncommunicable Diseases

**DOI:** 10.3928/24748307-20240520-01

**Published:** 2024-04

**Authors:** Ungsinun Intarakamhang, Khwanying Sriprasertpap, Araya Chiangkhong, Niwat Srisawasdi, Supitcha Wongchan, Piya Boocha

## Abstract

**Background:**

Noncommunicable diseases (NCDs) account for more than 75% of deaths in Thailand, which is higher than the global average of 71%.

**Objective:**

The aim of this study was to investigate the effects of the Digital Health Literacy (DHL) and Sufficient Health Behavior (SHB) Program on Thai working-age adults age 20 to 65 years with risk factors for NCDs (i.e., overweight and lacking physical activity), and compare the health literacy (HL) and SHB of participants living in urban and semi-urban areas at posttest.

**Methods:**

Using the lottery method, this one-group pretest-posttest quasi-experimental design randomly selected 200 participants and assigned them to two equally sized groups. The data were gathered through surveys with an item discrimination power between .20 and .86 and a reliability of 0.94 and were statistically analyzed using *t*-test and F-test.

**Key Results:**

The DHL and SHB Program comprises six sessions over a 12-week period, and activities designed to enhance knowledge of NCDs, HL, health communication, and health behavior modification. It was conducted by health care workers from urban and semi-urban public hospitals via Zoom using various digital toolkits such as YouTube, animations, infographics, role-play videos, clips, and e-books. At the posttest, the participants had higher HL (*t* = 2.67, *p* = .001) and SHB (*t* = 3.36, *p* = .001). There was a difference in SHB scores (*F* = 4.640, *p* = .032) between those living in urban and those in semi-urban areas, but no difference in HL scores (*F* = 1.436, *p* = .232).

**Conclusions:**

The DHL and SHB Program improved HL and SHB in Thai working-age adults with risk factors for NCDs in both urban and semi-urban communities. [***HLRP: Health Literacy Research and Practice*. 2024;8(2):e93–e101.**]

## Abstract (plain language summary)

**Plain Language Summary: **This study describes the implementation of a Digital Health Literacy and Sufficient Health Behavior Program via Zoom by health care workers in urban and semi-urban hospitals to effectively improve health literacy and sufficient health behavior among Thai working-age populations with risk factors for noncommunicable diseases. The Program was preliminarily shown to be extendable to other health care settings covered by the Internet.

The 10th Global Conference on Health Promotion conducted by the World Health Organization (WHO; 2017) showcased the need for people to take control of their lifestyles for longevity and to reduce premature mortality from noncommunicable diseases (NCDs) by 20%. NCDs account for 57 million deaths worldwide (i.e., 71% of all deaths) in 2016, and are responsible for 320,000 deaths (i.e., 75% of all deaths) in Thailand each year (Centers for Disease Control and Prevention, 2018).

In Thailand, the premature NCD mortality rate increased from 343.06 in 2009 to 355.30 per 100,000 population in 2013 (Department of Disease Control, 2020), with one-third of the NCD risk factors related to such mortality rates being lack of physical activity and weight control (Department of Disease Control, 2016). These two factors are particularly common among working-age Thai people, who are considered an NCD risk group. According to the International Diabetes Federation (2021), 1 in every 10 adults is living with diabetes, and 1 in every 3 people has hypertension worldwide (Centers for Disease Control and Prevention, 2018). In Thailand, 1 in every 11 working-age adults between ages 20 and 65 years suffer from diabetes, 50% of whom are not aware of their condition (Department of Disease Control, 2020). When comparing morbidity and mortality caused by four types of NCDs in 2021 in urban areas of Thailand, Bangkok showed the highest morbidity rate (1,270.36 per 100,000 population) and a mortality rate of 121.84 per 100,000 population, and these numbers are higher than those for the semi-urban province of Nonthaburi (mortality rate, 112.76 per 100,000 population; Department of Disease Control, 2023). However, NCDs can be prevented by three aspects of healthy lifestyle behaviors, namely physical health (e.g., weight control), mental health (e.g., emotion management), and social health (e.g., positive relationships with others) (Sarrami-Foroushani et al., 2014; Hacihasanoğlu & Gözüm, 2011; Pender et al., 2006; Rattanapun et al., 2009; Thiamwong et al., 2008). In the Policy and Strategy Section (2017), healthy lifestyle behaviors are defined as the “Thai way of life,” which in turn is based on the sufficiency economy philosophy that aims at enhancing people's immunity and potential to prevent NCDs by improving their health literacy (HL), which then helps people secure own health.

HL is a key factor influencing health behaviors and outcomes. It is also a mediator variable among personnel, environment, and health behavior-related variables (Brega et al., 2012; Hsu et al., 2014; Intarakamhang & Macaskill, 2018). A 2016 survey on HL conducted with Thai people aged 15 to 65 years revealed that most had poor health outcomes and HL, followed by fair and good HL (Intarakamhang & Kwanchuen, 2016). In response to these results, the National Health Development Plan of Thailand described the need to increase the population's HL by 25%, and to encourage Thai people to engage in appropriate health behaviors and improve behavioral determinants of health by 2024 (Ministry of Public Health, 2016).

The HL scale and the Sufficient Health Behavior (SHB) scale used in this study were developed according to relevant concepts proposed by the WHO (2017), which defines HL as “the cognitive and social skills that determine the ability of individuals to gain access to, understand, and use the information to promote and maintain good health.” Nutbeam (2000) divided HL into three levels, that is, functional literacy (e.g., basic reading), interactive literacy (e.g., cognitive literacy), and critical literacy (e.g., ability to analyze information to decide/act to constantly enhance/maintain health). Nutbeam (2008) also developed a framework for clinical care and public health development that suggests that HL affects people's health behaviors and that those with low HL have risk factors for diseases. In Thailand, six HL dimensions were developed through research syntheses, as follows: access, cognitive skills, communication skills, self-management, media literacy, and decision-making skills (Edwards et al., 2012; Intarakamhang & Macaskill, 2018; Manganello, 2008).

Meanwhile, the definition of HL used in the current study follows the propositions of Sørensen et al. (2012), who divided it into accessing (i.e., ability to seek/obtain health information); understanding (i.e., ability to comprehend health information); appraising (i.e., ability to explain/interpret/screen/evaluate health information); applying (i.e., ability to use health information to make health care decisions). The concept of SHB used in the current research was based on interview-based evidence on Thai people's perceptions collected through the qualitative study titled “Sufficient Health as Perceived by Thai villagers.” Arpanantikul (2018) defined sufficient health as living a moderate, reasonable, and careful life and regularly practicing self-care. Later, Intarakamhang et al. (2022) developed a 30-item, high-quality, reliable SHB scale, and demonstrated that HL had a positive influence on SHB, and predicted SHB at a 67.00% rate. In this study, sufficient health was defined as being healthy, having regular health check-ups, practicing self-care, sufficient living behaviors, and avoiding risks of disease, particularly NCDs. Thus, SHB was measured by sufficient living behaviors (e.g., living a simple life), safety behaviors (e.g., avoiding unsafe activities), and self-care behaviors (e.g., eating healthy).

Worldwide, interventions on HL and health behavior modification have generally treated the HL's concept as being related to lifelong learning or life lessons (Begoray & Kwan, 2012), and included HL in education curricula and literacy programs for local adults and older adults. Nevertheless, we should focus on developing people's critical HL through community-based health communication and enhancing their ability to face/adapt to change, specifically through using technology to facilitate community learning activities and to extend program applicability to various areas. Manafo and Wong (2012) suggested that HL interventions should be evidence-based and involve Internet-based communication, workshop activities, and standard self-assessment tools to enable individuals to take care of their health (Chiarella & Keefe, 2008; Friedman et al., 2006). Meanwhile, Vamos et al. (2020) developed an HL toolkit comprising lesson plans, videos, PowerPoint presentations, worksheet instructions, and grading rubrics for health instructors based on elements of HL. Before beginning the HL development process, it is important to ensure that instructors have appropriate HL development skills so that they can effectively foster learners' HL which, as confirmed by Vamos et al. (2016), can predict health behaviors and outcomes.

Researchers also demonstrated the effectiveness of promoting HL, health behaviors, and outcomes through online learning using digital media. Using formal and informal teaching methods, Conard (2019) showed that digital media-based learning (e.g., through videos and voice) was associated with HL and outcomes, as it provided the opportunity for individuals to be active participants in their own care and empowered them. Through digital media-based learning, participants in Conard's (2019) research could comfortably develop and share decision-making skills. This is consistent with Robbins and Dunn's (2019) research findings, wherein smartphone use to access health information—conceptually changing the focus from HL to digital health literacy (DHL)—and for health care professional–patient communication regarding health management could empower people to live healthier lives.

Therefore, this study aimed to investigate the effects of the digital-based DHL and SHB programs on Thai working-age people with risk factors for NCDs. The hypotheses were that participants would have higher average scores on HL and SHB at posttest, and there would be no average score differences by residency. The research question was as follows: can a digital program conducted by public health care professionals promote HL and SHB among Thai working-age people with risk factors for NCDs?

## Methods

### Setting and Sample

This study adopted a one-group pretest-posttest quasi-experimental design. The participants were Thai working-age people age 20 to 65 years. We chose this population because they can be regarded as more emotionally mature and have reasoning capabilities that support critical thinking. Working-age adults living in urban (Bangkok) and semi-urban (the five surrounding provinces) areas of Thailand were shown to have the highest NCD morbidity rates.

The sample size was determined using G*Power 3.1.9.4 and considering a significance level, power, and medium effect size of .05, .95, and .37, respectively, for two-tailed hypothesis tests (Buchner, 2023; Cohen, 1977). This yielded a sample of 97, which was increased by 3% to compensate for potential dropouts, resulting in 200 participants divided into two groups (urban and semi-urban) of 100 participants. Sample recruitment was conducted randomly using the lottery method from the target population that sought treatment at local health-promoting primary care hospitals in the study regions and expressed a desire to participate in the experiment.

The inclusion criteria were as follows: age 20 to 65 years and have risk factors for NCDs by being overweight or lacking physical activity (self-reported); living in urban or semi-urban areas of Thailand; able to participate online via computer or smartphone; able to participate in the whole experiment period. The exclusion criteria were suffering from NCDs during the experiment; inability to participate in the whole experiment period; and withdrawal from the study.

### Instruments

**Figure 1.**
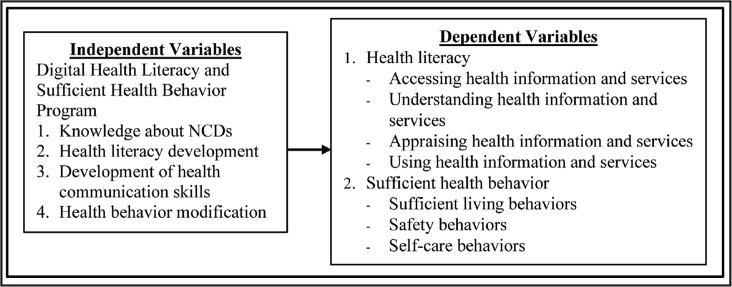
Research framework.

First, the experiment involved the DHL and SHB Program's application, which was developed based on the concepts of HL from Sørensen et al. (2012), SHB from Arpanantikul (2018), and learning management and behavior modification from Bandura (1986). The Program encompassed 6 weekly, 2-hour sessions conducted by health care workers via Zoom and that could be accessed through smartphone and a web-site, and various digital toolkits were implemented (e.g., You-Tube, animations, infographics, role-play videos, clips, and e-books). The Program's content validity was examined by three psychology and behavioral science experts, resulting in item-objective congruence index values between .66 and 1.00. The Program was also preliminarily tested with a sample group with similar characteristics to explore the appropriateness of its sequence, duration, and content, and identify flaws and problems that might occur during the experiment. Based on the data of this test, the Program was modified. Regarding research methods, the researchers developed an experimental setup wherein public health care professionals involved in the program were previously trained on HL skills, positive health communication skills, and behavior modification based on the sufficiency economy philosophy. During the experimental process, participants were required to complete a survey at the Behavioral Science Research Institute's website. Once they answered all survey questions, they received their results regarding HL and SHB levels. This served as a form of feedback to raise participants' awareness of their own HL and motivate their engagement in the learning activities of the online classes through Zoom. The research framework also used Bandura's (1986) learning management and behavior modification concepts to design activities as shown in **Figure 1** and the program activities are presented in **Table 1**.

**Table 1:**
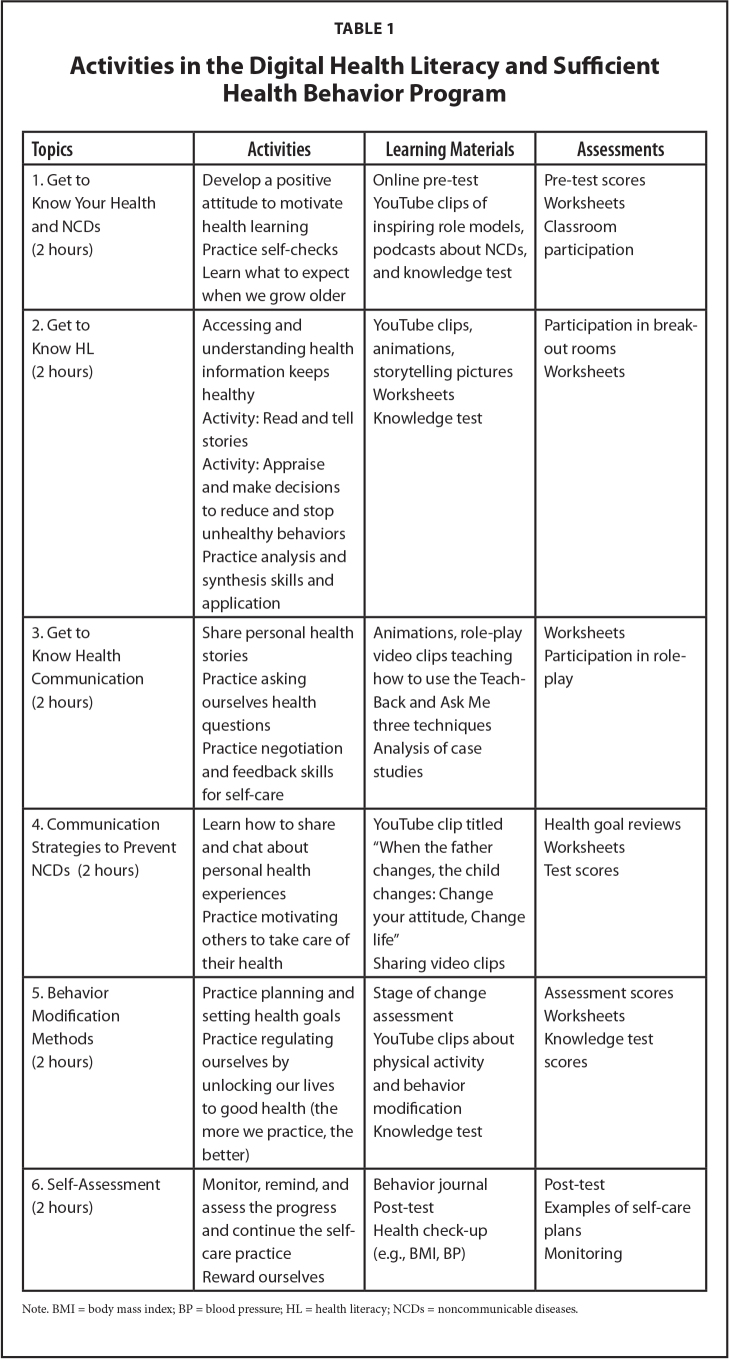
Activities in the Digital Health Literacy and Sufficient Health Behavior Program

**Topics**	**Activities**	**Learning Materials**	**Assessments**

1. Get to Know Your Health and NCDs (2 hours)	Develop a positive attitude to motivate health learning Practice self-checks Learn what to expect when we grow older	Online pre-test YouTube clips of inspiring role models, podcasts about NCDs, and knowledge test	Pre-test scores Worksheets Classroom participation

2. Get to Know HL (2 hours)	Accessing and understanding health information keeps healthy	YouTube clips, animations, storytelling pictures Worksheets Knowledge test	Participation in break-out rooms Worksheets
	Activity: Read and tell stories Activity: Appraise and make decisions to reduce and stop unhealthy behaviors Practice analysis and synthesis skills and application		

3. Get to Know Health Communication (2 hours)	Share personal health stories	Animations, role-play video clips teaching how to use the Teach-Back and Ask Me three techniques Analysis of case studies	Worksheets Participation in role-play
	Practice asking ourselves health questions		
	Practice negotiation and feedback skills for self-care		

4. Communication Strategies to Prevent NCDs (2 hours)	Learn how to share and chat about personal health experiences Practice motivating others to take care of their health	YouTube clip titled “When the father changes, the child changes: Change your attitude, Change life” Sharing video clips	Health goal reviews Worksheets Test scores

5. Behavior Modification Methods (2 hours)	Practice planning and setting health goals Practice regulating ourselves by unlocking our lives to good health (the more we practice, the better)	Stage of change assessment YouTube clips about physical activity and behavior modification Knowledge test	Assessment scores Worksheets Knowledge test scores

6. Self-Assessment (2 hours)	Monitor, remind, and assess the progress and continue the self-care practice Reward ourselves	Behavior journal Post-test Health check-up (e.g., BMI, BP)	Post-test Examples of self-care plans Monitoring

Note. BMI = body mass index; BP = blood pressure; HL = health literacy; NCDs = noncommunicable diseases.

Data collection at pretest and posttest was conducted using the DHL scale (28 items) and the SHB scale (30 items) developed by the researchers. As aforementioned, the HL scale was created based on Sørensen et al.'s (2012) four-dimension (i.e., accessing, understanding, appraising, and applying) conceptualization of HL, while the SHB scale was based and three elements of sufficient health (i.e., sufficient living behaviors, safety behaviors, and self-care behaviors) according to the Thai concept of sufficiency economy (Arpanantikul, 2018). Both scales were responded on a 5-point scale ranging from 1 (lowest) to 5 (highest), were examined for content validity, and preliminarily tested with 100 people who had similar characteristics to those of the experiment sample. The scales showed item-objective congruence index values between .66 and 1.00 and an acceptable quality level as indicated by the values of item discrimination power, reliability, and construct validity as shown in **Table 2**.

**Table 2:**
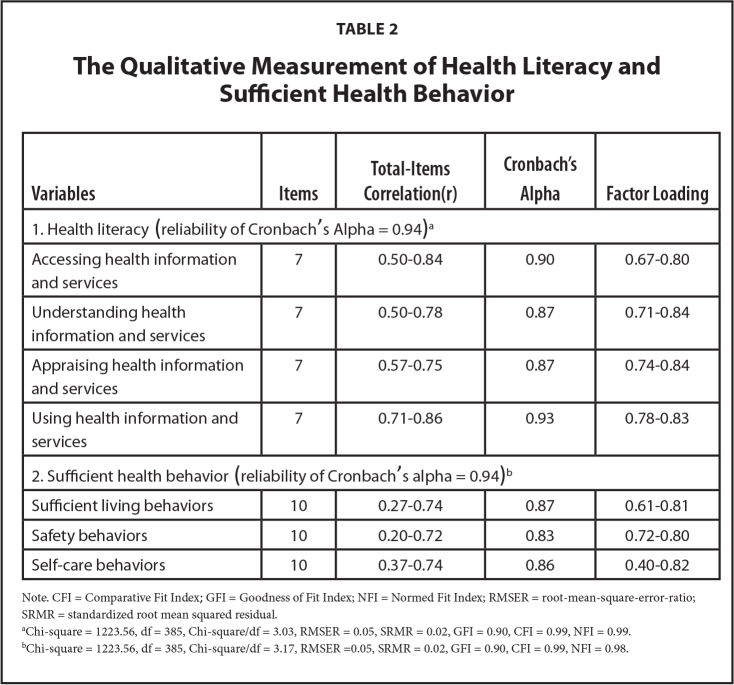
The Qualitative Measurement of Health Literacy and Sufficient Health Behavior

**Variables**	**Items**	**Total-Items Correlation(r)**	**Cronbach's Alpha**	**Factor Loading**
1. Health literacy (reliability of Cronbach 's Alpha = 0.94)^a^				
Accessing health information and services	7	0.50–0.84	0.90	0.67–0.80
Understanding health information and services	7	0.50–0.78	0.87	0.71–0.84
Appraising health information and services	7	0.57–0.75	0.87	0.74–0.84
Using health information and services	7	0.71–0.86	0.93	0.78–0.83
2. Sufficient health behavior (reliability of Cronbach 's alpha = 0.94)^b^				
Sufficient living behaviors	10	0.27–0.74	0.87	0.61–0.81
Safety behaviors	10	0.20–0.72	0.83	0.72–0.80
Self-care behaviors	10	0.37–0.74	0.86	0.40–0.82

Note. CFI = Comparative Fit Index; GFI = Goodness of Fit Index; NFI = Normed Fit Index; RMSER = root-mean-square-error-ratio; SRMR = standardized root mean squared residual.

a Chi-square = 1223.56, df = 385, Chi-square/df = 3.03, RMSER = 0.05, SRMR = 0.02, GFI = 0.90, CFI = 0.99, NFI = 0.99.

b Chi-square = 1223.56, df = 385, Chi-square/df = 3.17, RMSER =0.05, SRMR = 0.02, GFI = 0.90, CFI = 0.99, NFI = 0.98.

### Data Analysis

Data analyses involved examining the basic assumption for conducting *t*-tests and data normality using Kolmogorov-Smirnov test, analyzing basic sample characteristics using descriptive statistics (frequency, percentage, and standard deviation), and the difference in average scores between the two residency groups using *t*-test and *F*-test. We conducted ANCOVA to compare the average scores for HL and SHB between the urban and semi-urban groups at the posttest, with pretest scores as the covariate.

All participants provided informed consent prior to study onset and received information about study details. Confidentiality was ensured, and measures were taken to prevent potential harm to participants. This study was approved by the appropriate ethics review board (No. SWUEC-330/2021).

## Results

### Participants' Basic Characteristics

In the urban group (*n* = 100), 67% were female and 33% were male participants; 60%, 16%, and 14% were in the 20 to 30, 41 to 50, and 51 to 60 age groups, respectively; 67%, 18%, and 13% had a bachelor's degree or higher, a vocational certificate, and a high school certificate, respectively.

In the semi-urban group (*n* = 100), 72% were female and 28% were male participants, 66%, 13%, and 8% were in the 20 to 30, 51 to 60, and 41 to 50 age groups, respectively; 61%, 17%, and 15% had a bachelor's degree or higher, a high school certificate, and a vocational/high vocational certificate, respectively. The sample's basic data are presented in **Table 3**.

**Table 3:**
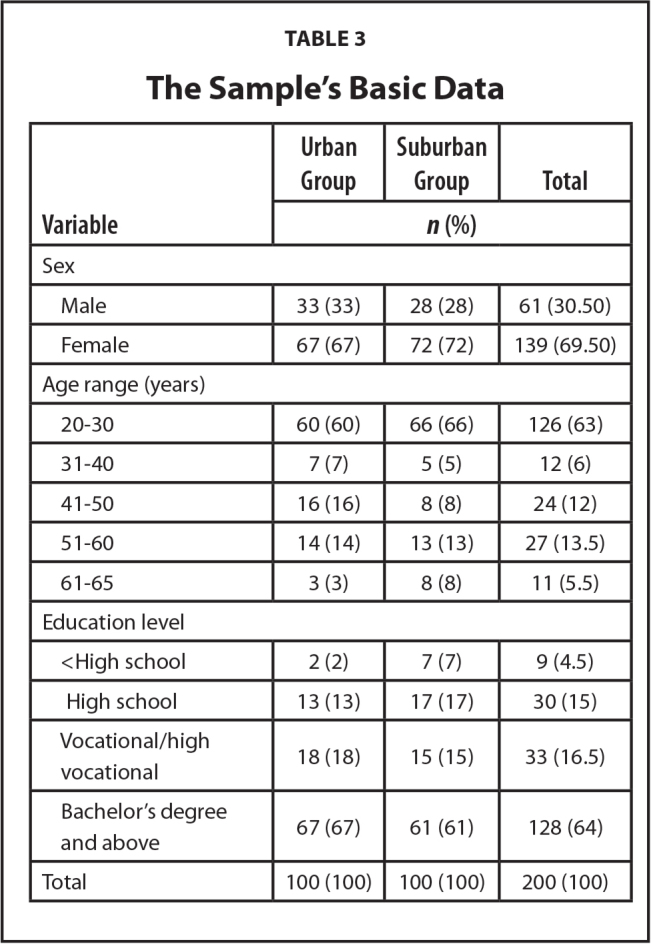
The Sample's Basic Data

**Variable**	**Urban Group**	**Suburban Group**	**Total**
	***n* (%)**		
Sex			
Male	33 (33)	28 (28)	61 (30.50)
Female	67 (67)	72 (72)	139 (69.50)
Age range (years)			
20–30	60 (60)	66 (66)	126 (63)
31–40	7 (7)	5 (5)	12 (6)
41–50	16 (16)	8 (8)	24 (12)
51–60	14 (14)	13 (13)	27 (13.5)
61–65	3 (3)	8 (8)	11 (5.5)
Education level			
<High school	2 (2)	7 (7)	9 (4.5)
High school	13 (13)	17 (17)	30 (15)
Vocational/high vocational	18 (18)	15 (15)	33 (16.5)
Bachelor's degree and above	67 (67)	61 (61)	128 (64)
Total	100 (100)	100 (100)	200 (100)

### Hypothesis Testing

Results demonstrated that the data were normally distributed. Thus, we examined differences in the sample's average scores.

### Urban Group

At posttest, participants had a significantly higher average HL score (*t* = 2.67, *p* < .05), and for the accessing, appraising, and applying subscales (*t* = 2.87, 2.27, and 2.86, respectively; *p* < .05). Nonsignificant differences were observed for the understanding subscale.

At posttest, participants showed a significantly higher average SHB score (*t* = 3.36, *p* < .05), and for the sufficient living behaviors, safety behaviors, and self-care behaviors subscales (*t* = 2.52, 3.87, and 3.00, respectively; *p* < .05) as shown in **Table 4**.

**Table 4:**
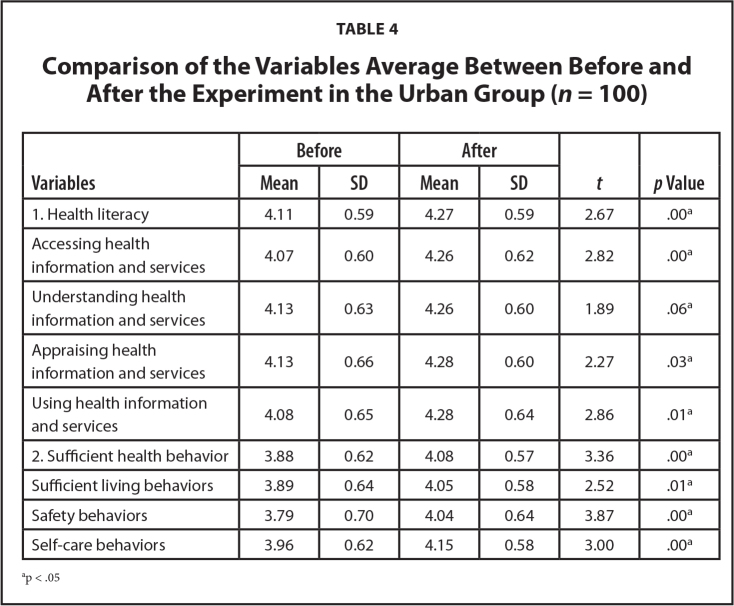
Comparison of the Variables Average Between Before and After the Experiment in the Urban Group (*n* = 100)

**Variables**	**Before**		**After**		***t***	***p* Value**
	**Mean**	**SD**	**Mean**	**SD**		
1. Health literacy	4.11	0.59	4.27	0.59	2.67	.00^a^
Accessing health information and services	4.07	0.60	4.26	0.62	2.82	.00^a^
Understanding health information and services	4.13	0.63	4.26	0.60	1.89	.06^a^
Appraising health information and services	4.13	0.66	4.28	0.60	2.27	.03^a^
Using health information and services	4.08	0.65	4.28	0.64	2.86	.01^a^
2. Sufficient health behavior	3.88	0.62	4.08	0.57	3.36	.00^a^
Sufficient living behaviors	3.89	0.64	4.05	0.58	2.52	.01^a^
Safety behaviors	3.79	0.70	4.04	0.64	3.87	.00^a^
Self-care behaviors	3.96	0.62	4.15	0.58	3.00	.00^a^

a p < .05

### Semi-Urban Group

At posttest, participants had a significantly higher average HL score (*t* = 2.65, *p* < .05), and for accessing, understanding, appraising, and applying (*t* = 2.55, 2.42, 2.34, and 2.67, respectively; *p* < .05).

At posttest, participants showed a significantly higher average SHB score (*t* = 3.00, *p* < .05), and for sufficient living behaviors, safety behaviors, and self-care behaviors (*t* = 3.16, 2.74, and 2.69, respectively; *p* < .05) as shown in **Table 5**.

**Table 5:**
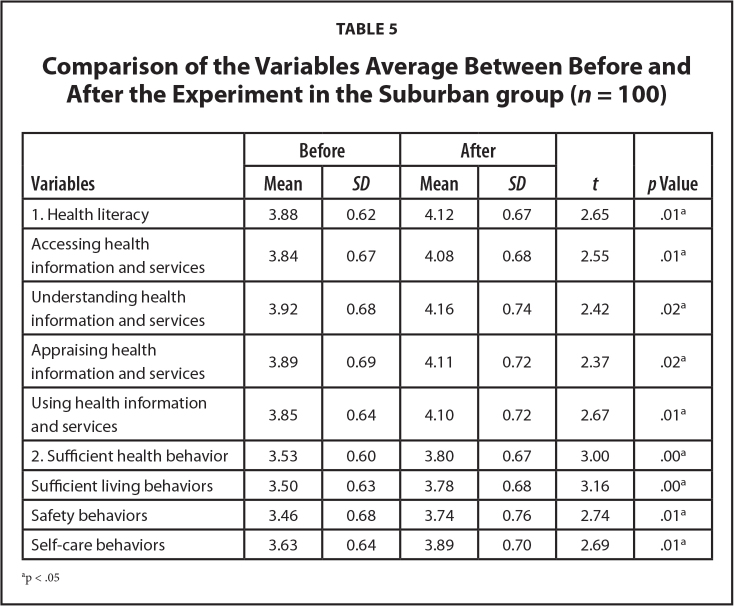
Comparison of the Variables Average Between Before and After the Experiment in the Suburban group (*n* = 100)

**Variables**	**Before**		**After**		***t***	***p* Value**
	**Mean**	***SD***	**Mean**	***SD***		
1. Health literacy	3.88	0.62	4.12	0.67	2.65	.01^a^
Accessing health information and services	3.84	0.67	4.08	0.68	2.55	.01^a^
Understanding health information and services	3.92	0.68	4.16	0.74	2.42	.02^a^
Appraising health information and services	3.89	0.69	4.11	0.72	2.37	.02^a^
Using health information and services	3.85	0.64	4.10	0.72	2.67	.01^a^
2. Sufficient health behavior	3.53	0.60	3.80	0.67	3.00	.00^a^
Sufficient living behaviors	3.50	0.63	3.78	0.68	3.16	.00^a^
Safety behaviors	3.46	0.68	3.74	0.76	2.74	.01^a^
Self-care behaviors	3.63	0.64	3.89	0.70	2.69	.01^a^

a p < .05

### Group Comparison

The results of ANCOVA (Analysis of Covariance) showed a nonsignificant difference between groups in average scores for HL, and a significant difference between groups in average scores for SHB (*p* < .05) as shown in **Table 6**.

**Table 6:**
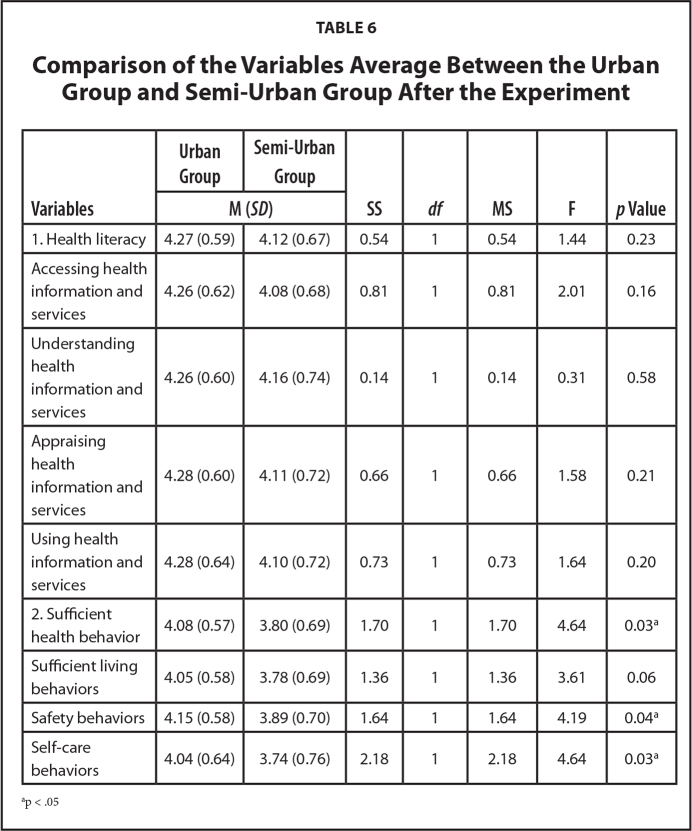
Comparison of the Variables Average Between the Urban Group and Semi-Urban Group After the Experiment

**Variables**	**Urban Group**	**Semi-Urban Group**	**SS**	***df***	**MS**	**F**	***p* Value**
	**M (*SD*)**						
1. Health literacy	4.27 (0.59)	4.12 (0.67)	0.54	1	0.54	1.44	0.23
Accessing health information and services	4.26 (0.62)	4.08 (0.68)	0.81	1	0.81	2.01	0.16
Understanding health information and services	4.26 (0.60)	4.16 (0.74)	0.14	1	0.14	0.31	0.58
Appraising health information and services	4.28 (0.60)	4.11 (0.72)	0.66	1	0.66	1.58	0.21
Using health information and services	4.28 (0.64)	4.10 (0.72)	0.73	1	0.73	1.64	0.20
2. Sufficient health behavior	4.08 (0.57)	3.80 (0.69)	1.70	1	1.70	4.64	0.03^a^
Sufficient living behaviors	4.05 (0.58)	3.78 (0.69)	1.36	1	1.36	3.61	0.06
Safety behaviors	4.15 (0.58)	3.89 (0.70)	1.64	1	1.64	4.19	0.04^a^
Self-care behaviors	4.04 (0.64)	3.74 (0.76)	2.18	1	2.18	4.64	0.03^a^

a p < .05

## Discussion

The DHL and SHB Program comprised activities designed to enhance individuals' knowledge about NCDs, HL, health communication, and health modification, and targeted Thai working-age people. The results of the overall effectiveness test of the program were consistent with the research hypotheses. After the experiment, the participants had higher levels of HL and SHB, and there was a difference in SHB scores between those living in urban and semi-urban areas, but no difference in HL scores. Thus, the Program was preliminarily shown to be effective and applicable by health care workers to improve HL and SHB among Thai working-age populations with risk factors for NCDs. These findings corroborate those in Benny et al.'s (2021) review of 131 experimental studies applying electronic HL models, showing that most digital health interventions are conducted to enhance the understanding, process, and actions related to health information via websites (51.9%) among those suffering from NCDs (43.5%) or mental health problems (19.8%), which can effectively increase HL. Furthermore, most studies focused on HL development (73.2%), followed by digital (14.5%), media (3%), and information (0.7%), with some covering both HL and DHL (5.3%). The effects of HL on health behaviors and outcomes have also been confirmed (Brega et al., 2012; Hsu et al., 2014).

At the posttest, participants in the semi-urban group had a higher average score for all aspects of HL and SHB than at the pretest. In the urban group, there was no difference in the understanding subscale of HL between pre-test and posttest. The reason for the divergent results by residency may be that the participants living in semi-urban areas had an unhurried lifestyle and more self-learning time than those living in urban areas. Moreover, participants in the semi-urban group were mostly female participants and younger, and these two demographic characteristics may be more conducive to better digital social media use skills. Therefore, the DHL portion of the program was more suitable for semi-urban participants. Meanwhile, participants in the urban group showed a higher level of safety and self-care behaviors than those in the semi-urban group at posttest. This may be explained by environmental factors in urban areas being more supportive of engagement in exercise in public areas, healthy food markets, accident prevention, and care systems. Therefore, the SHB portion of the program was more suitable for urban area participants.

These findings differ from those in Intarakamhang and Macaskill's (2021) research, wherein a program based on positive psychology affected the HL and health behaviors of Thai families with risk factors for NCDs. They also depict that urban area working-age participants had lower HL (vs. living in rural areas) and did not differ in health behaviors. Meanwhile, our results corroborate those described by Jafree et al.'s (2023) study among 360 women in Pakistan, wherein a health care worker-led DHL intervention influenced the health awareness of infection prevention behavior. Andersson (2018) confirmed that the online approach enables patients to access health information, and exchange information with health providers to monitor, and improve people's education and health behaviors, indicating that technology use in educational settings is increasing.

Regarding practical implications, health care providers working in semi-urban and rural communities in the central region of Thailand are urged to extend the application of this Program to health services in these areas. This may help enhance DHL and SHB for working-age populations in Thailand. Regarding future research, we suggest scholars to conduct experimental research with control groups to explore more effective approaches for developing HL and SHB, which may support NCD risk reduction. They could also design action research aimed at promoting HL and SHB, or qualitative research among participants whose levels of HL and SHB remained the same after an intervention.

## Limitations and Conclusions

The study was conducted in Bangkok and the surrounding provinces. Therefore, the findings may not be representative of all health care providers and populations in the country, as the characteristics and circumstances of individuals in rural areas or different regions could differ significantly. Almost all participants were female because this study did not control for extraneous variables. The objective of this preliminary study was to test the Program's applicability to semi-urban areas. Therefore, comparisons with a control group and follow-up after the experiment were not performed. A randomized controlled trial with long-term follow-up should be designed in future research.

The DHL and SHB Program was preliminarily shown to be usable by urban and semi-urban health care workers to improve HL and SHB among the Thai working-age population. The scales developed may also be useful for assessments before and after conducting the DHL and SHB Program, or for other HL and health behavior interventions for working-age groups with risk factors for NCDs.
